# Artificial intelligence-driven work systems and employee burnout: a systematic review of mechanisms, moderators, and a dynamic meta-system framework

**DOI:** 10.3389/fpsyg.2026.1922281

**Published:** 2026-10-02

**Authors:** Zicheng Fang, Liangliang Han, Te Ma

**Affiliations:** 1 Business School, Liaoning University, Shenyang, Liaoning, China; 2 Emergency Management Teaching and Research Department, Party School of Dalian Committee of C.P.C., Dalian, Liaoning, China

**Keywords:** algorithmic management, artificial intelligence, employee burnout, job demands–resources model, systematic review, technostress

## Abstract

**Background:**

The rapid integration of artificial intelligence (AI) into workplace systems is transforming job design and employee experiences. While AI promises efficiency gains, it also creates a paradox by simultaneously reducing workload and increasing psychological strain, leading to divergent effects on burnout.

**Objective:**

This study systematically reviews evidence on the relationship between AI exposure and employee burnout and develops the AI as Meta-System (AIMS) framework.

**Methods:**

Following PRISMA 2020, four bibliographic databases and Google Scholar were searched from inception to 31 March 2026. Forty-three peer-reviewed primary empirical studies were included: 20 measured burnout or an established burnout dimension, and 23 examined mechanisms, moderators, proxy outcomes, measurement, or implementation conditions. Methodological quality was appraised using the Mixed Methods Appraisal Tool at the criterion level.

**Results:**

Findings varied according to the functional role of AI and the study design. Assistive AI was generally associated with lower burnout or exhaustion in randomized and pre–post healthcare studies, whereas monitoring and algorithmic-control exposures were associated with greater burnout or related psychosocial strain in observational studies. Perceptual AI exposure produced direct, indirect, and null associations through pathways involving job stress, job insecurity, work–family interference, perceived organizational support, and organizational commitment. Three randomized studies provided the strongest evidence for assistive interventions. Evidence for nonlinear effects was limited to supporting proxy outcomes and did not directly establish an inverted U-shaped AI–burnout relationship.

**Conclusion:**

Artificial intelligence is not inherently harmful or beneficial; its associations with burnout depend on its functional role, implementation, and employee appraisal. The AIMS framework integrates these pathways and identifies propositions requiring longitudinal and experimental testing.

## 1 Introduction

The rapid diffusion of artificial intelligence (AI)–driven work systems is fundamentally transforming how work is organized, executed, and evaluated across industries. Organizations increasingly rely on algorithmic management, real-time monitoring, and AI-enabled decision support to enhance productivity, efficiency, and innovation. While these systems promise substantial operational gains, a growing body of empirical research suggests that they may also generate unintended negative consequences for employee psychological health (Stamate et al., 2021). Empirical studies indicate that AI awareness, adoption, and algorithmic control may be associated with job stress, job insecurity, poorer well-being, or burnout (Kong et al., 2021; Kim and Lee, 2024; Dong et al., 2025; Wang and Zhou, 2025). Conversely, ambient documentation and AI-tailored interventions have generally been associated with lower burnout or exhaustion when they reduce documentation and workload demands (Afshar et al., 2025; Baek and Cha, 2025; Chowdhury et al., 2026; Olson et al., 2025; You et al., 2025). Related quality-improvement evidence has also examined clinicians’ perceptions of work burden, burnout, and job satisfaction following ambient AI documentation (Albrecht et al., 2025). This mixed evidence suggests that AI does not have a uniform effect on employee well-being, but instead produces context-dependent outcomes. Review and conceptual sources are used in this article to establish the broader theoretical context but are not counted among the 43 primary empirical studies included in the systematic-review synthesis.

This divergence gives rise to a central paradox. AI can simultaneously reduce workload by automating routine tasks while increasing psychological strain through new demands such as continuous monitoring, uncertainty, and accelerated work pace. Empirical findings illustrate this contradiction. Workers exposed to automation risk may experience lower immediate stress yet report poorer health and reduced job satisfaction (Nazareno and Schiff, 2021), while AI adoption increases burnout indirectly through job stress mechanisms (Kim and Lee, 2024). Broader evidence has similarly linked automation probability with psychosocial working conditions and worker health (Cheng et al., 2021), while employee perceptions of smart technologies, AI, robotics, and algorithms have been examined as an important aspect of anticipated workplace change (Brougham and Haar, 2018). AI-related identity threat, job-automation awareness, and perceived replacement risk may also increase cognitive job insecurity and undermine employee well-being (Gull et al., 2023; Chung et al., 2025; Lingmont and Alexiou, 2020). AI adoption has also been examined in relation to psychological contracts, job engagement, and employee trust (Braganza et al., 2021). These findings raise a critical question: under what conditions does AI reduce burnout, and when does it amplify it?

Although prior research on technology and work has established links between digitalization and burnout, primarily through technostress and job demand frameworks, AI represents a qualitatively different class of technology. Unlike traditional systems, AI is adaptive, opaque, and capable of allocating tasks and monitoring performance in real time (Zerilli et al., 2022; Zheng et al., 2025). These characteristics introduce novel stressors, including algorithmic opacity, techno-unpredictability, and perceived loss of control (Issa et al., 2024; Kumar et al., 2024). Importantly, while existing reviews have examined AI and burnout in specific sectors such as healthcare (Dave et al., 2026) and have provided multilevel perspectives on AI in organizations (Bankins et al., 2024), no synthesis has integrated AI-specific mechanisms across occupations within a dynamic, multi-pathway theoretical framework. Consequently, the literature does not yet provide an integrated account of the conditions under which AI exposure is associated with burnout-related outcomes across occupational contexts.

This gap has significant theoretical and practical implications. Without a clear understanding of the mechanisms, moderators, and boundary conditions linking AI to burnout, organizations risk implementing AI systems in ways that undermine employee well-being and long-term productivity. Burnout is associated with substantial organizational costs, including turnover, reduced performance, and increased healthcare burden (Shanafelt et al., 2017; Martinez et al., 2025). Evidence suggests that poorly implemented AI systems, characterized by excessive monitoring, inadequate training, or unrealistic expectations, may exacerbate stress and disengagement, whereas human-centered implementations may mitigate these risks (Meduri et al., 2024). Thus, understanding how AI shapes burnout is both a theoretical imperative and a managerial necessity.

To address these limitations, we conceptualize AI not solely as a job demand or a job resource but as a work system that may simultaneously alter demands, resources, and control. The Job Demands–Resources framework provides a useful theoretical foundation for examining these domains (Bakker et al., 2023), while the proposed AIMS framework makes their concurrent and interactive operation explicit in AI-enabled work. The AIMS framework is therefore presented as a theory-building extension of existing models rather than as evidence that established occupational-health frameworks are inadequate.

Building on this premise, this study develops a dynamic, multi-pathway, contingency-based framework to explain how, when, and why AI-driven work systems influence employee burnout. It synthesizes interdisciplinary evidence to identify key mechanisms, including technostress, job insecurity, emotional strain, dependency, skill degradation, AI-related fear of missing out, work–family interference, and organizational-support pathways (Högemann et al., 2025; Méndez-Suárez et al., 2026; Zheng and Zhang, 2025; Wang and Zhou, 2025). It further examines moderators and boundary conditions, including organizational support, self-efficacy, and system design characteristics, while identifying critical gaps such as the lack of longitudinal designs and AI-specific measurement.

The primary conceptual contribution of this paper is the AI as Meta-System (AIMS) framework. The framework treats AI as potentially operating as a demand amplifier, a resource provider, and a control system and organizes proposed pathways involving workload, identity-related concerns, and temporal adaptation. It also specifies candidate individual and organizational moderators and proposes a dynamic adaptation trajectory requiring direct longitudinal testing. Rather than establishing causal mechanisms, the AIMS framework integrates the observed associations and generates research propositions for future experimental, longitudinal, and multi-wave studies.

## 2 Methods

### 2.1 Protocol and reporting standards

This systematic review was conducted in accordance with the PRISMA 2020 guidelines (Page et al., 2021) to ensure transparency, rigor, and reproducibility. A review protocol was developed *a priori* to define the research objectives, eligibility criteria, and analytical procedures. Due to the interdisciplinary nature of the study, spanning organizational behavior, information systems, and occupational health, the protocol was not registered in PROSPERO; however, all procedures were predefined to minimize selection and reporting bias.

### 2.2 Search strategy

A comprehensive literature search was conducted across four bibliographic databases, Web of Science, Scopus, PubMed/MEDLINE, and APA PsycINFO, supplemented by Google Scholar and backward and forward citation searching. The search strategy combined keywords related to artificial intelligence, workplace contexts, and burnout outcomes using Boolean operators. The core search string included terms such as “artificial intelligence,” “machine learning,” “algorithmic management,” and “automation,” combined with “burnout,” “emotional exhaustion,” “stress,” and “well-being,” as well as “employee,” “worker,” and “professional.”

Backward and forward citation tracking was also conducted to ensure comprehensive coverage, and database-specific adaptations of the search syntax were applied. Complete database-specific search strategies, limits, and search fields are provided in Supplementary Table S1.

### 2.3 Eligibility criteria

Studies were included if they met the following criteria: (1) an empirical quantitative, qualitative, mixed-methods, randomized, or non-randomized design; (2) publication as a peer-reviewed journal article; (3) examination of an identifiable AI-driven, algorithmic, robotic, automation, or machine-learning system in a workplace context; (4) inclusion of employees, workers, professionals, or occupational groups; and (5) reporting either (a) burnout or an established burnout dimension or (b) an occupational psychological outcome used specifically to examine a mechanism, moderator, boundary condition, or implementation factor theoretically relevant to burnout.

Eligible outcomes were classified *a priori* into two groups. Direct or core-dimension outcomes included burnout, emotional exhaustion, work exhaustion, interpersonal disengagement, depersonalization, and professional efficacy. Supporting outcomes included technostress, job stress, job insecurity, work–family interference, occupational well-being, career sustainability, psychological health, and related mechanisms or boundary conditions. Supporting outcomes were synthesized separately and were not interpreted as equivalent to a validated burnout endpoint.

Studies were excluded if they were reviews, conceptual articles, editorials, protocols, commercial reports, conference proceedings, non-peer-reviewed sources, studies of non-working populations, studies without an identifiable workplace AI or automation exposure, or studies without an eligible occupational psychological outcome. Only English-language reports were considered.

### 2.4 Study selection

All identified records were imported into EndNote (X9) for reference management. Duplicate records were removed automatically and verified manually. Titles and abstracts were screened independently by two reviewers, and disagreements were resolved through discussion or consultation with a third reviewer.

The search initially yielded 1,248 records. After removal of 312 duplicate records, 936 records underwent title and abstract screening, of which 742 were excluded. The full texts of 194 reports were assessed for eligibility, and 151 reports were excluded because they did not meet the empirical-design, population, exposure, outcome, publication-status, or reporting criteria. A total of 43 peer-reviewed primary empirical studies were included in the final synthesis, comprising 20 studies measuring burnout or an established burnout dimension and 23 supporting empirical studies addressing mechanisms, moderators, proxy outcomes, measurement, or implementation conditions. The complete study-selection process is presented in Figure 1, while the operational exclusion criteria and potentially eligible reports excluded after full-text assessment are provided in Supplementary Tables S2A,B, respectively.

**Figure 1:**
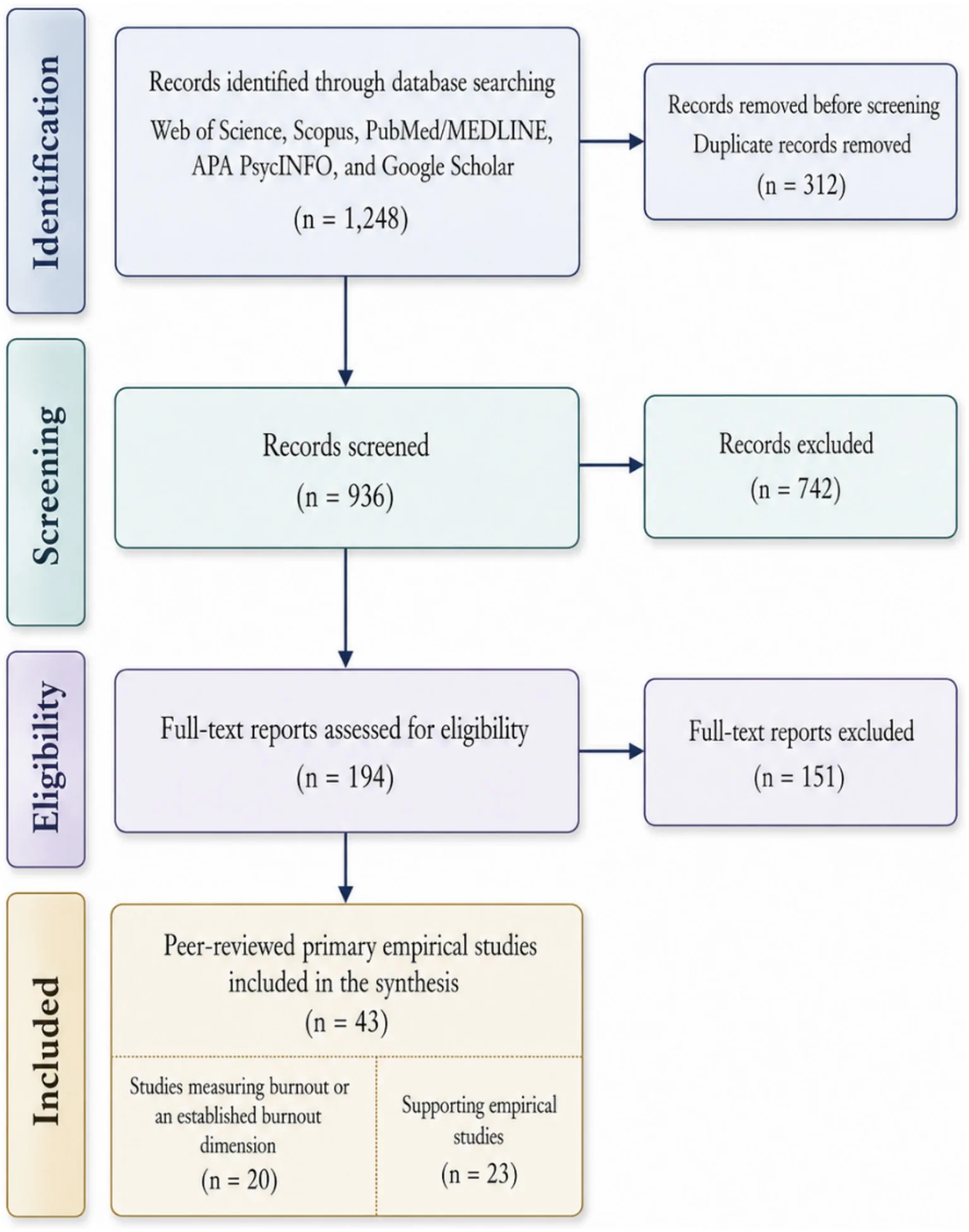
PRISMA 2020 flow diagram of study identification, screening, eligibility assessment, and inclusion. The database search identified 1,248 records. After removal of 312 duplicates, 936 records were screened and 194 full-text reports were assessed for eligibility. Following the exclusion of 151 full-text reports, 43 peer-reviewed primary empirical studies were included in the final synthesis. Of these, 20 measured burnout or an established burnout dimension, and 23 provided supporting empirical evidence on mechanisms, moderators, proxy outcomes, measurement, or implementation conditions.

### 2.5 Data extraction

A standardized data extraction protocol was applied to all included studies. Extracted information included study characteristics (author, year, country, and industry), sample size and population, study design, type and functional role of the AI system, outcome or construct measured, measurement instrument, and reported relationships between AI exposure and burnout, established burnout dimensions, or supporting occupational psychological outcomes. Where available, effect sizes, including standardized coefficients, odds ratios, correlations, confidence intervals, and statistical significance, were recorded.

In addition, studies were systematically coded to identify underlying mechanisms (e.g., technostress, job insecurity, identity threat), moderating variables (e.g., organizational support, self-efficacy), and contextual boundary conditions. The complete study-level extraction matrix is provided in Supplementary Tables S3A,B. Studies measuring burnout or an established burnout dimension are reported in Supplementary Table S3A, whereas studies addressing mechanisms, moderators, proxy outcomes, measurement, or implementation conditions are reported separately in Supplementary Table S3B.

### 2.6 Quality assessment

Methodological quality was assessed using the Mixed Methods Appraisal Tool (MMAT 2018; Hong et al., 2018), which permits design-specific appraisal of qualitative, randomized, non-randomized, descriptive, and mixed-methods studies. The two MMAT screening questions were applied to all studies, followed by the five criteria corresponding to the relevant design category. Two reviewers completed the appraisal independently, and disagreements were resolved through discussion. Criterion-level judgments of yes, no, or cannot tell are reported in Supplementary Table S4. Consistent with MMAT guidance, no aggregate numerical quality score was calculated. Recurrent limitations involved sample representativeness, nonresponse reporting, attrition, self-reported outcomes, and control of confounding, whereas randomized, longitudinal, and repeated-measures studies generally provided stronger evidence.

### 2.7 Data synthesis

Due to substantial heterogeneity in study designs, AI operationalizations, and burnout measures, a quantitative meta-analysis was not feasible. Instead, a theory-driven narrative synthesis was conducted.

The synthesis proceeded in three stages. First, empirical findings were organized according to the core dimensions of the proposed AIMS framework: demands, resources, and control. Second, a thematic coding approach was used to identify recurring mechanisms and moderators across studies. Third, cross-study comparisons were conducted to identify consistent patterns, contradictions, and boundary conditions, which informed the development of a dynamic, multi-pathway theoretical framework. Direct or core-dimension burnout evidence and supporting evidence were synthesized separately to prevent proxy psychological outcomes from being interpreted as equivalent to burnout.

## 3 Results

### 3.1 Characteristics of included studies

A total of 43 peer-reviewed primary empirical studies met the inclusion criteria. Twenty studies measured burnout or an established burnout dimension, whereas 23 examined mechanisms, moderators, proxy outcomes, measurement, or implementation conditions relevant to AI-related employee well-being. The evidence base comprised three randomized controlled studies, 19 quantitative non-randomized studies, 16 quantitative descriptive studies, three qualitative studies, and two mixed-methods studies. Sample sizes ranged from 15 participants in qualitative interviews to 27,252 workers in a large European survey.

AI exposure was operationalized in three broad functional roles: assistive systems, including ambient documentation and AI-tailored interventions; monitoring or control systems, including algorithmic management and automated task allocation; and perceptual exposures, including AI awareness, replacement concerns, trust, or perceived threat. Table 1 summarizes all 20 studies measuring burnout or an established burnout dimension. Supplementary Table S3A provides the corresponding study-level extraction information, while Supplementary Table S3B reports the 23 supporting studies addressing mechanisms, proxy outcomes, measurement, or implementation conditions.

**Table 1:** Characteristics of studies measuring burnout or an established burnout dimension.

Study	Country/context	Design and sample	AI exposure and role	Burnout outcome	Principal finding
Afshar et al. (2025)	USA; healthcare	Pragmatic stepped-wedge randomized trial; 66 practitioners	Ambient AI scribe; assistive	Work exhaustion and interpersonal disengagement	Mean change −0.44 points, 95% CI −0.62 to −0.25; *p* < 0.001
Baek and Cha (2025)	South Korea; nursing	Three-group single-blind RCT; 120 nurses	AI-tailored intervention; assistive	Personal, work-related, and client-related burnout	Client-related burnout: *F* = 7.725, *p* = 0.001; personal burnout: *F* = 10.967, *p* < 0.0001
Chowdhury et al. (2026)	USA; outpatient healthcare	Open-label randomized 2 × 2 crossover trial; 160 randomized, 136 analyzed	Two ambient AI scribe platforms; assistive	Copenhagen Burnout Inventory	Both platforms reduced personal and work-related burnout relative to baseline; between-platform burnout differences were not meaningful
Cho et al. (2024)	South Korea; nursing	Multiphase single-arm intervention-development study; 300 nurses completed the optimization phase	AI-based tailored mobile intervention; assistive	Copenhagen Burnout Inventory and job stress	Burnout decreased after the first program and decreased further after the second program
Olson et al. (2025)	USA; healthcare	Multicenter pre–post study; 263 clinicians	Ambient AI scribe; assistive	Single-item professional burnout	Burnout decreased from 51.9 to 38.8%; OR = 0.26, 95% CI 0.13–0.54
Misurac et al. (2025)	USA; healthcare	Pre–post observational study; 38 enrolled, 35 completed	Ambient AI-generated notes; assistive	Stanford Professional Fulfillment Index burnout	Median burnout score decreased from 4.16 to 3.16, *p* = 0.005; prevalence decreased from 69 to 43%
Liu et al. (2024)	China; radiology	Nationwide cross-sectional survey; 6,726 radiologists	AI use in radiology; use/perceptual exposure	Maslach Burnout Inventory	AI use was associated with burnout; adjusted OR = 1.20, 95% CI 1.10–1.30
Dong et al. (2025)	China; gig economy	Cross-sectional survey; 953 delivery riders	Algorithmic management; monitoring/control	Maslach Burnout Inventory–General Survey	Multiple dimensions of algorithmic management were positively associated with burnout
Kong et al. (2021)	China; hospitality	Cross-sectional SEM; 432 hotel employees	AI awareness; perceptual threat	Job burnout	AI awareness was positively related to job burnout
Kim and Lee (2024)	South Korea; mixed occupations	Three-wave survey; 416 professionals	AI adoption; perceptual exposure	Burnout and job stress	No significant direct AI–burnout association; significant indirect pathway through job stress; AI self-efficacy buffered the pathway
Ali et al. (2024)	Pakistan; mixed occupations	Cross-sectional survey; 300 employees	AI and automation exposure	Burnout, stress, anxiety, and job insecurity	AI exposure correlated with burnout, *r* = 0.54; stress, *r* = 0.72; and anxiety, *r* = 0.58; all *p* < 0.01
AlOqaily et al. (2025)	Jordan; industrial sector	Cross-sectional mediation study; 149 employees	AI implementation in HRM; assistive/organizational	Job burnout and work–life balance	Work–life balance mediated the association between AI implementation and lower burnout
Gao et al. (2025)	China; gig economy	Cross-sectional survey; 475 workers	Perceived algorithmic control; monitoring/control	Burnout as mediator	Burnout mediated the adverse association between algorithmic control and service performance; transparency weakened the indirect pathway
Chuang et al. (2025)	Country not reported; mixed occupations	Three-wave survey; 600 AI-using employees	AI technostress, AI efficacy, and generative AI	Exhaustion	AI technostress increased exhaustion, whereas AI efficacy reduced exhaustion
Thorpe et al. (2026)	Australia; mixed occupations	Cross-sectional survey; 197 workers	Generative-AI use; assistive/perceptual	Burnout, job demands, and job control	No statistically significant direct association between generative-AI use and burnout
Meduri et al. (2024)	Healthcare and IT contexts	Cross-sectional survey; 320 workers	Human-centered AI workload management; assistive	Employee burnout/well-being	Training, personalization, and feedback were associated with more favorable burnout-related outcomes
You et al. (2025)	USA; healthcare	Implementation surveys at two academic health systems; 1,430 clinicians enrolled	Ambient documentation technology; assistive	Professional Fulfillment Index burnout	Follow-up respondents reported significant reductions in burnout and improved documentation-related well-being; follow-up response was limited
Stults et al. (2025)	USA; healthcare	Pre–post quality-improvement study; 100 clinicians, 57 paired surveys	Ambient AI documentation; assistive	Mini-Z burnout	Burnout decreased from 42.1 to 35.1%, but the change was not statistically significant, *p* = 0.12
Zheng and Zhang (2025)	China; private sector	Cross-sectional survey; 303 employees	AI awareness; perceptual threat	Emotional exhaustion	AI awareness positively predicted emotional exhaustion; job insecurity and work interference with family formed a serial indirect pathway
Wang and Zhou (2025)	China; higher education	Cross-sectional survey; 434 university teachers	AI awareness; perceptual threat	Job burnout	AI awareness positively predicted burnout; perceived organizational support and organizational commitment formed significant mediating pathways

Table presents the 20 included studies that measured burnout or an established burnout dimension. The complete evidence inventory for all 43 included primary empirical studies is provided in Supplementary Tables S3A,B. Supporting outcomes such as technostress, job insecurity, job satisfaction, career sustainability, and occupational well-being are reported separately and are not interpreted as equivalent to a validated burnout endpoint. AI, artificial intelligence; CBI, Copenhagen Burnout Inventory; CI, confidence interval; HRM, human resource management; MBI, Maslach Burnout Inventory; OR, odds ratio; RCT, randomized controlled trial; SEM, structural equation modeling.

### 3.2 Synthesis of quantitative findings

The quantitative findings, summarized in Table 2, indicated a role-dependent rather than uniform pattern. Studies measuring burnout or an established burnout dimension were analyzed separately from studies reporting supporting occupational psychological outcomes, including technostress, occupational stress, job insecurity, job satisfaction, and general well-being.

**Table 2:** Selected quantitative findings for direct burnout, core burnout dimensions, and supporting proxy outcomes.

Study	Sample/design	AI exposure	Quantitative finding	Direction	Outcome classification
Afshar et al. (2025)	66; stepped-wedge RCT	Ambient AI scribe	Mean change in work exhaustion/interpersonal disengagement: −0.44, 95% CI −0.62 to −0.25; *p* < 0.001	Lower strain	Core burnout dimensions
Baek and Cha (2025)	120; three-group RCT	AI-tailored intervention	Client-related burnout: *F* = 7.725, *p* = 0.001; personal burnout: *F* = 10.967, *p* < 0.0001	Lower burnout	Direct burnout
Chowdhury et al. (2026)	160 randomized; 136 analyzed; randomized crossover trial	Two ambient AI scribes	Both platforms reduced personal and work-related burnout relative to baseline; no meaningful between-platform burnout difference	Lower burnout	Direct/core; randomized
Cho et al. (2024)	300; single-arm intervention	AI-tailored mobile intervention	Burnout decreased after program 1, *t* = 7.012, *p* < 0.001, and decreased further after program 2, *t* = 2.811, *p* = 0.01	Lower burnout	Direct burnout; uncontrolled
Olson et al. (2025)	263; multicenter pre–post	Ambient AI scribe	Burnout decreased from 51.9 to 38.8%; OR = 0.26, 95% CI 0.13–0.54	Lower burnout	Direct burnout
Misurac et al. (2025)	38 enrolled, 35 completed; pre–post	Ambient AI notes	Median score decreased from 4.16 to 3.16, *p* = 0.005; prevalence decreased from 69 to 43%	Lower burnout	Direct burnout
Liu et al. (2024)	6,726; cross-sectional	AI use in radiology	Adjusted OR for burnout = 1.20, 95% CI 1.10–1.30	Higher burnout	Direct burnout
Dong et al. (2025)	953; cross-sectional	Algorithmic management	Multiple algorithmic-management dimensions were positively associated with burnout	Higher burnout	Direct burnout
Kong et al. (2021)	432; cross-sectional SEM	AI awareness	AI awareness was positively related to job burnout	Higher burnout	Direct burnout
Kim and Lee (2024)	416; three-wave study	AI adoption	Direct AI–burnout association was not significant; indirect effect through job stress was significant	Indirect/mixed	Direct and indirect burnout evidence
Ali et al. (2024)	300; cross-sectional	AI and automation exposure	Burnout *r* = 0.54; stress *r* = 0.72; anxiety *r* = 0.58; all *p* < 0.01	Higher strain	Direct burnout and related outcomes
AlOqaily et al. (2025)	149; cross-sectional mediation	AI implementation in HRM	Work–life balance mediated the association with lower burnout	Lower through mediator	Direct burnout
Gao et al. (2025)	475; cross-sectional moderated mediation	Algorithmic control	Burnout mediated the adverse performance pathway; transparency weakened the indirect association	Higher burnout pathway	Burnout-mediated observational evidence
Chuang et al. (2025)	600; three-wave study	AI technostress and AI efficacy	AI technostress increased exhaustion; AI efficacy reduced exhaustion	Bidirectional	Core burnout dimension
Thorpe et al. (2026)	197; cross-sectional	Generative-AI use	No statistically significant direct association with burnout	Null	Direct burnout
You et al. (2025)	1,430 enrolled; implementation surveys	Ambient documentation technology	Follow-up respondents reported significant reductions in burnout; response rates were low	Lower burnout	Direct burnout; implementation evidence
Stults et al. (2025)	100 enrolled, 57 paired surveys; pre–post	Ambient AI documentation	Burnout decreased from 42.1 to 35.1%, *p* = 0.12	Lower but NS	Direct burnout
Zheng and Zhang (2025)	303; cross-sectional serial mediation	AI awareness	Positive association with emotional exhaustion; serial indirect pathway through job insecurity and work interference with family	Higher exhaustion	Core burnout dimension
Wang and Zhou (2025)	434; cross-sectional chain mediation	AI awareness	Positive association with burnout; significant mediating pathways through organizational support and commitment	Higher burnout with protective pathways	Direct burnout
Payá Castiblanque and Pizzi (2024)	27,252; cross-sectional	AI monitoring and task allocation	Associated with time pressure, overload, reduced autonomy, and stress-related outcomes	Higher strain	Supporting psychosocial proxy
Koch and Lodefalk (2025)	Approximately 20,000 per wave; repeated cross-sectional	AI/robot exposure	Not associated with higher stress and negatively associated with increased stress in several models	Lower/no higher stress	Supporting stress proxy
Wu et al. (2026)	Labor-force secondary data; sample NR	AI exposure/intensity	Inverted U-shaped association with job satisfaction; turning point varied by skill level	Nonlinear	Supporting job-satisfaction proxy

Direct outcomes comprise burnout or an established burnout dimension, including emotional exhaustion, work exhaustion, and interpersonal disengagement. Supporting proxy outcomes are included to explain mechanisms, boundary conditions, or adjacent psychological consequences and are not interpreted as equivalent to burnout. CI, confidence interval; HRM, human resource management; NR, not reported; NS, not statistically significant; OR, odds ratio; RCT, randomized controlled trial; SEM, structural equation modeling.

Assistive AI systems were generally associated with lower burnout or exhaustion when they reduced documentation, workload, or cognitive demands. Randomized and pre–post healthcare studies reported reductions in burnout or established burnout dimensions following the use of ambient AI scribes or AI-tailored interventions (Afshar et al., 2025; Baek and Cha, 2025; Chowdhury et al., 2026; Cho et al., 2024; Misurac et al., 2025; Olson et al., 2025; You et al., 2025). However, these findings were not uniform. Stults et al. (2025), for example, reported a reduction in burnout from 42.1 to 35.1%, but the difference was not statistically significant. Moreover, several implementation studies lacked concurrent control groups, and some had low follow-up response or unpaired pre- and post-implementation samples.

Monitoring and algorithmic-control exposures were generally associated with greater burnout or related psychosocial strain. Algorithmic management was positively associated with burnout among delivery riders (Dong et al., 2025), and burnout mediated the adverse association between perceived algorithmic control and service performance among gig workers (Gao et al., 2025). Large-scale European evidence further linked AI-enabled monitoring and task allocation with time pressure, reduced autonomy, and stress-related outcomes (Payá Castiblanque and Pizzi, 2024). Perceptual AI exposure produced more heterogeneous results. AI awareness was positively associated with burnout or emotional exhaustion in Kong et al. (2021), Zheng and Zhang (2025), and Wang and Zhou (2025); Kim and Lee (2024) identified an indirect pathway through job stress rather than a significant direct association; and Thorpe et al. (2026) found no statistically significant direct relationship between generative-AI use and burnout. Three randomized studies provided the strongest evidence concerning assistive interventions, but all were conducted in healthcare contexts. Evidence for nonlinear effects was limited to supporting proxy outcomes, including the inverted U-shaped association between AI exposure and job satisfaction reported by Wu et al. (2026), and did not directly establish a nonlinear AI–burnout relationship.

### 3.3 Mechanisms linking AI to burnout

The mechanisms and mediating pathways identified across the included studies are summarized in Table 3. The evidence indicated that AI exposure was seldom associated with burnout through a single pathway. Instead, the included studies reported associations involving AI-related demands, resources, control conditions, and psychological or organizational mechanisms. In Chuang et al. (2025), AI technostress was positively associated with exhaustion, whereas AI efficacy was negatively associated with exhaustion. Kim and Lee (2024) reported a significant indirect association between AI adoption and burnout through job stress. Zheng and Zhang (2025) reported a serial indirect association through job insecurity and work interference with family, while Wang and Zhou (2025) identified indirect associations involving perceived organizational support and organizational commitment. Work–life balance was examined as a potential resource pathway in AlOqaily et al. (2025), and Gao et al. (2025) reported an indirect association between algorithmic control and service performance through burnout. These mediation findings should be interpreted in light of the observational designs used in most of the relevant studies.

**Table 3:** Mechanisms and mediating pathways relevant to AI-related burnout and occupational strain.

Study	Mechanism or mediator	Proposed or tested pathway	Interpretation
Chuang et al. (2025)	AI technostress and exhaustion	AI technostress → exhaustion → work and family outcomes	Exhaustion represents an established burnout dimension
Kim and Lee (2024)	Job stress	AI adoption → job stress → burnout	Significant indirect pathway; direct AI–burnout association was not significant
Zheng and Zhang (2025)	Job insecurity and work interference with family	AI awareness → job insecurity → work interference with family → emotional exhaustion	Serial indirect pathway to an established burnout dimension
Wang and Zhou (2025)	Perceived organizational support and organizational commitment	AI awareness → organizational-resource perceptions → burnout	Organizational support and commitment formed significant chain-mediating pathways
AlOqaily et al. (2025)	Work–life balance	AI implementation → work–life balance → burnout	Work–life balance mediated the association with lower burnout
Gao et al. (2025)	Burnout	Perceived algorithmic control → burnout → service performance	Burnout mediated the adverse association between algorithmic control and performance
Dong et al. (2025)	Reduced autonomy and algorithmic control demands	Algorithmic management → reduced autonomy/intensified control → burnout	Monitoring and control conditions were associated with higher burnout
Gull et al. (2023)	Cognitive job insecurity	AI-related identity threat → cognitive job insecurity → poorer well-being	Supporting threat pathway; burnout was not directly measured
Högemann et al. (2025)	Dependency, ambiguity, and skill degradation	Generative-AI use → AI-specific stressors → occupational strain	Qualitative supporting mechanism; burnout was not directly measured
Issa et al. (2024)	Techno-unpredictability, appraisal, and coping	AI unpredictability → distress or eustress depending on appraisal and coping	Demonstrates that the same AI demand may produce divergent psychological responses
Méndez-Suárez et al. (2026)	AI FoMO	Skill devaluation, autonomy loss, and AI supervision → AI FoMO	Supporting configurational mechanism; not a direct burnout endpoint
Zheng et al. (2025)	Autonomy erosion and surveillance precarity	AI-enabled HRM → autonomy loss, surveillance, bias, and personalized discontentment	Qualitative/contextual pathway relevant to occupational strain

Mechanisms derived from studies that did not directly measure burnout are identified as supporting pathways. Their outcomes should not be interpreted as equivalent to burnout. AI, artificial intelligence; FoMO, fear of missing out; HRM, human resource management.

The supporting and qualitative studies identified additional AI-specific mechanisms, including dependency, regulatory ambiguity, skill degradation, autonomy loss, surveillance precarity, and AI-related fear of missing out (Högemann et al., 2025; Méndez-Suárez et al., 2026; Zheng et al., 2025). These constructs help explain how AI may generate occupational strain, but they were not interpreted as direct burnout outcomes unless burnout or an established burnout dimension was explicitly measured.

### 3.4 Moderators and boundary conditions

The individual, organizational, and system-level moderators and boundary conditions identified across the included studies are summarized in Table 4. These factors help explain why similar forms of AI exposure may be associated with different psychological outcomes across employees and organizational settings.

**Table 4:** Moderators and boundary conditions relevant to AI-related burnout and occupational well-being.

Study	Moderator or boundary condition	Level	Reported role
Kim and Lee (2024)	AI self-efficacy	Individual	Weakened the adverse indirect pathway from AI adoption to burnout through job stress
Jin et al. (2024)	Resilience	Individual	Buffered the adverse pathway involving AI awareness, job stress, and employee well-being
Chung et al. (2025)	Career resilience	Individual	Weakened the association between AI awareness and job insecurity
Kong et al. (2023)	Protean career orientation	Individual/career	Shaped the relationship among trust in AI, employee–AI collaboration, and career sustainability
Wang and Zhou (2025)	Perceived organizational support and organizational commitment	Organizational	Formed protective chain-mediating pathways between AI awareness and burnout
Lingmont and Alexiou (2020)	Organizational culture	Organizational	Authoritarian culture strengthened the association between job-automation awareness and job insecurity
Gao et al. (2025)	Algorithmic transparency	System/organizational	Weakened the adverse indirect pathway from algorithmic control through burnout
Meduri et al. (2024)	Training, personalization, feedback, and implementation quality	Organizational/implementation	Associated with more favorable burnout-related and well-being outcomes
Issa et al. (2024)	Appraisal and coping	Individual/contextual	Determined whether AI unpredictability generated distress or eustress
Arboh et al. (2025)	Informal learning and employee appraisal	Individual/organizational	Contributed to demand and resource pathways between AI awareness and workplace well-being

Several factors in table were examined in relation to supporting outcomes such as job insecurity, career sustainability, or workplace well-being rather than burnout itself. They are therefore interpreted as boundary conditions relevant to the AIMS framework, not as confirmed moderators of every AI–burnout relationship. AI, artificial intelligence.

At the individual level, AI self-efficacy weakened the adverse indirect pathway from AI adoption to burnout through job stress (Kim and Lee, 2024). Resilience and career resilience buffered adverse pathways involving AI awareness, job stress, job insecurity, and employee well-being (Jin et al., 2024; Chung et al., 2025). At the organizational level, perceived organizational support and organizational commitment formed protective pathways between AI awareness and burnout (Wang and Zhou, 2025). Organizational culture also shaped employee responses: authoritarian culture strengthened the association between job-automation awareness and job insecurity (Lingmont and Alexiou, 2020). At the system and implementation levels, algorithmic transparency weakened the adverse indirect pathway associated with algorithmic control (Gao et al., 2025), while training, personalization, and implementation support were associated with more favorable employee outcomes in human-centered AI systems (Meduri et al., 2024). Appraisal and coping further determined whether AI unpredictability was experienced as distress or eustress (Issa et al., 2024).

### 3.5 Qualitative findings

Three qualitative or qualitative-dominant studies provided detailed evidence concerning employees’ experiences of AI-enabled work. Högemann et al. (2025) identified dependency, regulatory ambiguity, monitoring shifts, and perceived skill degradation among young professionals using generative AI. Kumar et al. (2024) described an automation–augmentation paradox in which AI and machine learning could simultaneously generate technostress and provide opportunities for socio-technical coping. Zheng et al. (2025) identified autonomy erosion, surveillance precarity, algorithmic bias, and personalized discontentment in AI-enabled human-resource systems.

These findings complement the quantitative evidence by explaining how AI-related demands, resources, and control structures are experienced by employees. However, because these studies primarily examined technostress, well-being, or broader occupational experiences, their findings should be interpreted as explanatory evidence concerning mechanisms and implementation conditions rather than as quantitative estimates of AI effects on burnout. Complete characteristics of these and the other supporting studies are reported in Supplementary Table S3B.

### 3.6 Heterogeneity and contradictory evidence

The apparent heterogeneity in the evidence can be partly explained by differences in AI role, outcome definition, study design, and implementation context. Assistive systems were generally associated with lower burnout or exhaustion when they reduced documentation or workload demands, but the magnitude and statistical significance of change varied across implementation studies. Monitoring and algorithmic-control exposures were associated with greater burnout or related psychosocial strain, although the relevant evidence was predominantly cross-sectional. Perceptual AI exposure produced positive, indirect, and null findings depending on appraisal, self-efficacy, organizational support, resilience, and job insecurity. Thus, the evidence supports a role-dependent pattern but not a universal beneficial or harmful effect of AI.

### 3.7 Risk of bias and methodological quality

The evidence base was dominated by observational designs, particularly cross-sectional surveys, which limited causal inference and increased vulnerability to common-method bias. Three randomized studies (Afshar et al., 2025; Baek and Cha, 2025; Chowdhury et al., 2026) and several longitudinal or pre–post studies provided stronger temporal evidence, although attrition, self-report measurement, low follow-up response, and the absence of concurrent controls remained important limitations in some implementation studies. Criterion-level MMAT judgments for all 43 studies are reported in Supplementary Table S4.

## 4 Discussion

### 4.1 Summary of key findings

This systematic review indicates that the association between AI-driven work systems and employee burnout depends on the functional role, implementation, and appraisal of the technology. Assistive systems were generally associated with lower burnout or exhaustion when they reduced documentation and workload demands, particularly in healthcare implementation studies. Monitoring and algorithmic-control systems were associated with greater burnout or psychosocial strain through intensified demands and reduced autonomy. Perceptual exposure, including AI awareness and replacement concerns, produced direct, indirect, and null associations through pathways involving job stress, job insecurity, work–family interference, perceived organizational support, organizational commitment, and resilience. Because most studies were observational, these patterns should be interpreted as associations rather than universal causal effects.

The review identified technostress, exhaustion, job insecurity, work–family interference, work–life balance, organizational support, and autonomy as recurrent pathways. AI-specific dependency, ambiguity, skill degradation, and FoMO emerged as additional potential stress mechanisms. Evidence for a nonlinear AI–burnout relationship remained limited. Wu et al. (2026) reported an inverted U-shaped association for job satisfaction, which was classified as a supporting proxy outcome rather than a direct burnout endpoint. The proposed shock–adjustment–mastery trajectory should therefore be interpreted as a theoretical proposition requiring direct longitudinal testing.

### 4.2 Theoretical contributions

#### 4.2.1 The AIMS model: AI as a meta-system beyond JD–R

The findings support a reconceptualization of AI as a meta-system that simultaneously reshapes job demands, resources, and control, extending the Job Demands–Resources (JD–R) model. Figure 2 presents the conceptual AI as Meta-System (AIMS) framework, in which AI is theorized to reshape job demands, job resources, and control structures simultaneously. These pathways may be associated with burnout through mechanisms including technostress, exhaustion, job insecurity, and emotional labor, while individual and organizational conditions may modify their strength or direction. The pathways shown in Figure 2 represent theory-derived relationships rather than causal effects established across all included studies.

**Figure 2:**
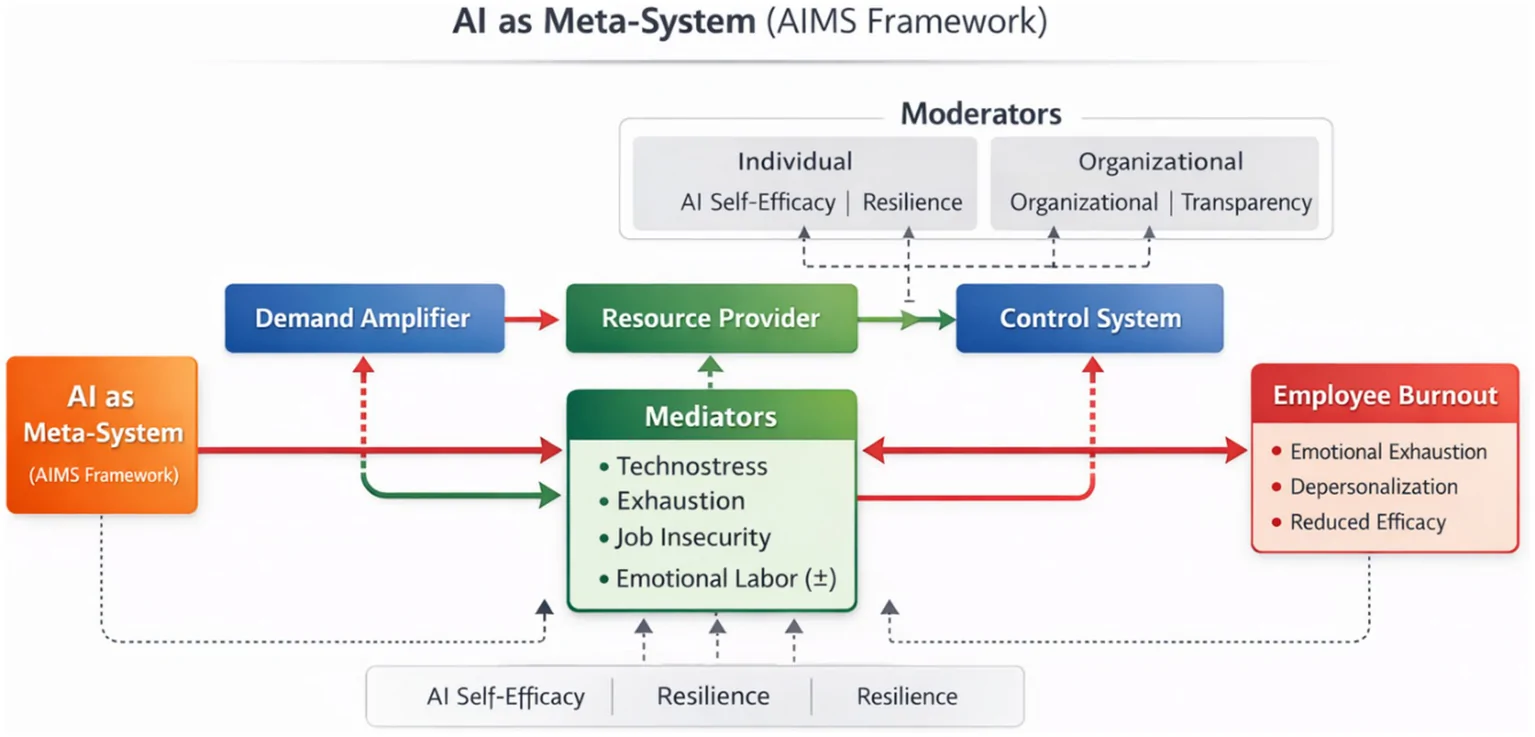
The AI as Meta-System (AIMS) conceptual framework. The framework conceptualizes AI as operating through three functional pathways: a demand-amplifying pathway that may be associated with higher burnout, a resource-providing pathway that may be associated with lower burnout, and a control pathway that may be associated with higher burnout or related psychosocial strain. Potential mechanisms, including technostress, exhaustion, job insecurity, and emotional labor, may transmit these associations, while individual and organizational conditions, including AI self-efficacy, resilience, organizational support, and transparency, may modify their strength or direction. The arrows represent theory-derived propositions and should not be interpreted as causal effects established across all included studies.

Empirical evidence illustrates this dual role. Empirical findings are consistent with this dual pattern. Chuang et al. (2025) reported that AI-related technostress was positively associated with emotional exhaustion (*γ* = 0.33), whereas AI efficacy was negatively associated with exhaustion (*γ* = −0.16). Similarly, Kim and Lee (2024) identified a significant indirect association between AI adoption and burnout through job stress, while the direct association was not statistically significant. These findings are consistent with multiple psychological pathways linking AI exposure to burnout-related outcomes but do not, by themselves, establish universal causal effects.

The AIMS framework extends JD–R by introducing three key elements: (1) simultaneous influence on demands, resources, and control; (2) non-linear pathways; and (3) dynamic adaptation over time. This shifts the conceptualization of work from task execution to continuous meta-cognitive regulation, where employees must interpret and respond to algorithmic systems.

#### 4.2.2 A role-based typology: assistive, monitoring, and perceptual AI

A central contribution of this review is the introduction of a role-based typology that reconciles conflicting findings in the literature. Distinguishing between assistive, monitoring, and perceptual AI clarifies why prior studies have reported divergent effects.

Assistive AI was generally associated with lower burnout or exhaustion when it reduced documentation, workload, or cognitive demands, particularly in healthcare intervention and implementation studies (Afshar et al., 2025; Baek and Cha, 2025; Chowdhury et al., 2026; Olson et al., 2025; You et al., 2025). Monitoring and algorithmic-control exposures were associated with greater burnout or related psychosocial strain through time pressure, reduced autonomy, surveillance, and intensified control (Dong et al., 2025; Gao et al., 2025; Payá Castiblanque and Pizzi, 2024). Perceptual exposure produced more heterogeneous findings and operated through mechanisms such as job stress, job insecurity, work–family interference, organizational support, and self-efficacy (Kim and Lee, 2024; Zheng and Zhang, 2025; Wang and Zhou, 2025). This typology advances prior research by explicitly linking AI functionality to psychological outcomes, rather than treating AI as a homogeneous construct.

#### 4.2.3 Dynamic and non-linear pathways

The included evidence does not directly establish an inverted U-shaped temporal trajectory between AI exposure and employee burnout. Wu et al. (2026) identified a nonlinear association between AI exposure and job satisfaction, but job satisfaction was treated as a supporting proxy outcome rather than a burnout measure. The proposed shock–adjustment–mastery curve is therefore presented as a conceptual model rather than an empirical estimate. It proposes that initial uncertainty and learning demands may increase strain, while subsequent adaptation, training, system familiarity, and organizational support may reduce strain over time.

AI-specific longitudinal evidence remains limited. Existing multi-wave, randomized, and pre–post studies generally cover relatively short implementation periods and do not test the complete proposed trajectory. Direct examination of within-person burnout before implementation, during adjustment, and after sustained use is therefore required. Figure 3 presents this theory-derived three-phase trajectory as a conceptual illustration requiring direct longitudinal testing.

**Figure 3:**
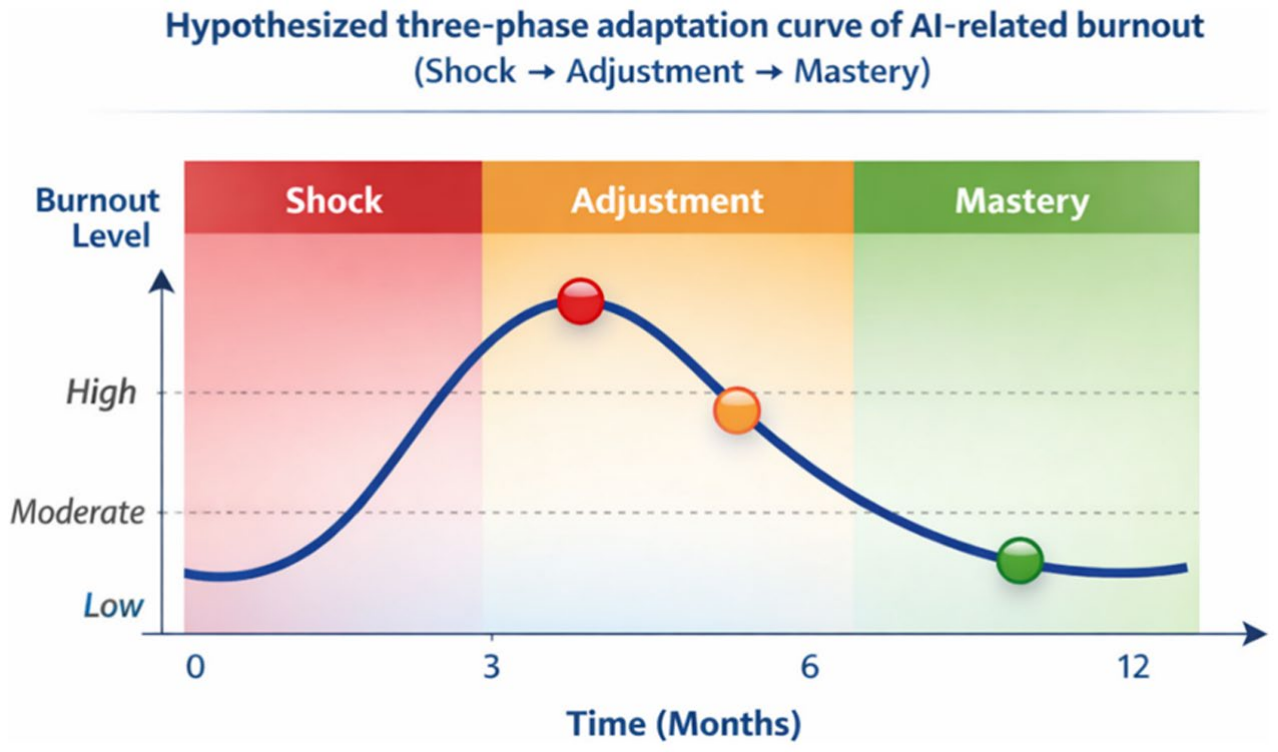
Proposed shock–adjustment–mastery trajectory of AI-related employee burnout. The figure is a theory-derived conceptual illustration rather than an empirical estimate. It proposes that strain may initially increase after AI implementation, peak during adjustment, and subsequently decline as employees develop competence and receive adequate organizational support. The timing, shape, and direction of the trajectory may differ according to AI role, implementation quality, job design, and individual resources and require direct longitudinal testing.

#### 4.2.4 Mechanisms and moderators

The review identified both established and emerging mechanisms relevant to AI-related burnout. Established pathways included technostress and exhaustion (Chuang et al., 2025), job stress (Kim and Lee, 2024), job insecurity and work interference with family (Zheng and Zhang, 2025), work–life balance (AlOqaily et al., 2025), and perceived organizational support and organizational commitment (Wang and Zhou, 2025). Emerging mechanisms included dependency, skill degradation, regulatory ambiguity, surveillance precarity, and AI-related FoMO (Högemann et al., 2025; Zheng et al., 2025; Méndez-Suárez et al., 2026).

Individual-level boundary conditions included AI self-efficacy, resilience, career resilience, and protean career orientation (Kim and Lee, 2024; Jin et al., 2024; Chung et al., 2025; Kong et al., 2023). Organizational and system-level conditions included perceived organizational support, organizational commitment, organizational culture, implementation quality, and algorithmic transparency (Meduri et al., 2024; Gao et al., 2025; Wang and Zhou, 2025; Lingmont and Alexiou, 2020). Complementary evidence also highlights trust, threat, and protean orientation in employee–AI collaboration and career sustainability (Duong et al., 2026), the role of generative AI in employees’ work-goal progress (Zhang et al., 2026), and the relationship between leadership AI awareness, hindrance/challenge stressors, and employee voice (Zhou and Lyu, 2025).

#### 4.2.5 Research propositions derived from the AIMS framework

The following propositions are theory-derived deductions from the AIMS framework and were not tested in the present systematic review.

*P1*: AI-driven job demands, including monitoring intensity, cognitive overload, and replacement concerns, are expected to be positively associated with employee burnout through technostress and job insecurity.

*P2*: AI-driven job resources, including automation of routine work and decision support, are expected to be negatively associated with burnout when they reduce workload and cognitive demands.

*P3*: AI-driven control mechanisms, including algorithmic monitoring and automated task allocation, are expected to be positively associated with burnout through reduced perceived autonomy.

*P4*: Professional identity threat is expected to mediate the association between perceived AI replacement risk and emotional exhaustion.

*P5*: AI self-efficacy is expected to weaken adverse associations between AI-driven demands and burnout.

*P6*: Perceived organizational support is expected to weaken adverse associations between AI exposure and burnout.

*P7*: AI-related burnout may follow nonlinear adaptation trajectories over time, with their direction and shape contingent on implementation quality, learning, job redesign, and organizational support.

*P8*: Algorithmic transparency is expected to weaken adverse associations between AI monitoring and burnout when it is accompanied by explanation, employee voice, and meaningful recourse.

### 4.3 Practical implications

The findings should be interpreted as design and implementation guidance rather than evidence that one form of AI will produce the same outcome in every organization. Assistive systems may reduce burnout or exhaustion when they demonstrably reduce documentation, workload, and cognitive demands. Organizations should therefore evaluate changes in workload and burnout before and after implementation rather than assuming that automation automatically improves well-being.

Monitoring and algorithmic-control systems require particular caution because observational studies associated these exposures with greater psychosocial strain, time pressure, reduced autonomy, or burnout. Where monitoring is necessary, organizations should provide transparent decision rules, employee voice, appeal or recourse mechanisms, clear accountability, and limits on data collection. These recommendations are also consistent with broader organizational research emphasizing the implications of transparency for privacy, organizational learning, operational control, and motivation (Bernstein, 2012; Brandes and Darai, 2017).

Training, personalization, technical support, and realistic performance expectations may improve implementation outcomes. Organizations should also monitor job insecurity, work–family interference, AI self-efficacy, resilience, and organizational support because these factors may shape employee responses. Burnout imposes substantial organizational costs, but future research should directly quantify whether responsible AI implementation reduces these costs over time.

### 4.4 Limitations and future research

Several limitations should be considered. First, the evidence base was dominated by observational and cross-sectional studies, limiting causal inference and increasing vulnerability to common-method and self-report bias. Three randomized studies were identified, but they were all conducted in healthcare and primarily examined assistive AI systems. Their findings may not generalize to monitoring, algorithmic-control, or non-healthcare contexts.

Second, direct comparisons of AI-based monitoring and human supervision using validated burnout outcomes were not identified. Gu et al. (2025) compared algorithmic and human supervision but was excluded from the systematic-review sample because it reported performance rather than an eligible psychological outcome. Future studies should compare these supervisory arrangements using longitudinal or experimental designs and validated occupational psychological endpoints.

Third, AI-specific longitudinal evidence remains insufficient to establish a shock–adjustment–mastery trajectory. Future studies should use repeated within-person measurements before implementation, during initial adjustment, and after sustained use. Fourth, cross-national and cross-occupational comparisons remain limited despite likely differences in regulation, labor-market conditions, organizational culture, and employee voice.

Fifth, measurement remains fragmented. Cross-national measurement research further underscores the importance of measurement invariance in burnout assessment (De Beer et al., 2020). Burnout instruments were not always AI-specific, and supporting outcomes such as technostress, job insecurity, job satisfaction, and well-being were operationalized inconsistently. Contextual scale-development studies outside the included AI-specific sample indicate growing interest in technology-related burnout measurement, but no comprehensive instrument currently integrates AI-specific demands, resources, control, identity threat, and adaptation.

Finally, experimental evidence concerning transparency, recourse, organizational support, resilience, and human-versus-algorithmic supervision remains limited. Future research should test these moderators directly rather than inferring their effects from cross-sectional associations.

## 5 Conclusion

AI was not uniformly associated with either beneficial or adverse burnout outcomes. Across the 20 studies measuring burnout or an established burnout dimension, assistive systems, examined predominantly in healthcare settings, were generally associated with lower burnout or exhaustion, whereas monitoring and algorithmic-control exposures were generally associated with greater burnout in observational studies. Perceptual AI exposures produced positive, indirect, and null associations. The 23 supporting studies clarified potential mechanisms, moderators, and implementation conditions but were not treated as direct burnout evidence. The AIMS framework organizes these findings into theory-derived pathways involving demands, resources, and control. Longitudinal, cross-occupational, and experimental studies are required before causal or temporal conclusions can be established.

## Data Availability

The datasets presented in this study can be found in online repositories. The names of the repository/repositories and accession number(s) can be found in the article/Supplementary material.
