# Supplementary material for: Artificial intelligence-driven work systems and employee burnout: a systematic review of mechanisms, moderators, and a dynamic meta-system framework
Source: Front Psychol. 2026 Oct 2;17:1922281. doi: 10.3389/fpsyg.2026.1922281 (PMC13672190; doi:10.3389/fpsyg.2026.1922281)
Supplement: Supplementary file 1 [file Table_1.DOCX]

**SUPPLEMENTARY MATERIAL**

**Artificial Intelligence-Driven Work Systems and Employee Burnout:
A Systematic Review of Mechanisms, Moderators, and a Dynamic Meta-System Framework**

Tables S1-S4 provide database-specific reproducible search syntax, full-text exclusion criteria and eligibility decisions, study-level characteristics of the 43 peer-reviewed primary empirical studies, and criterion-level methodological appraisal. Table S3A contains 20 studies measuring burnout or an established burnout dimension; Table S3B contains 23 empirical studies addressing mechanisms, moderators, proxy outcomes, measurement, or implementation conditions. Reviews, conceptual papers, commercial reports, conference proceedings, non-worker studies, and studies without an identifiable AI, algorithmic, robotic, or automation exposure are not included in the 43-study count. Table S4 reports MMAT 2018 judgments for all 43 included studies. NR indicates not reported; Y, yes; N, no; and CT, cannot tell from the published report.

**Supplementary Table S1. Database-specific reproducible search syntax and limits**

Search period: database inception to 31 March 2026. The strategies operationalize the AI, workplace, and burnout-related outcome domains reported in Section 2.2 of the manuscript.

| **Database/source** | **Platform** | **Search string** | **Limits** | **Search fields/details** |
| --- | --- | --- | --- | --- |
| Web of Science Core Collection | Clarivate Web of Science | TS=(("artificial intelligence" OR "machine learning" OR "generative AI" OR GenAI OR "large language model*" OR "algorithmic management" OR "algorithmic control" OR "algorithmic monitoring" OR automation OR robot*) AND (employee* OR worker* OR workforce OR workplace OR occupation* OR professional*) AND (burnout OR "emotional exhaustion" OR depersonalization OR "professional efficacy" OR technostress OR "job stress" OR "work stress" OR wellbeing OR "well-being" OR "work-life balance" OR "job insecurity")) | English; Article or Early Access; database inception to 31 March 2026 | Topic-field search covering title, abstract, author keywords, and Keywords Plus. |
| Scopus | Elsevier Scopus | TITLE-ABS-KEY(("artificial intelligence" OR "machine learning" OR "generative AI" OR GenAI OR "large language model*" OR "algorithmic management" OR "algorithmic control" OR "algorithmic monitoring" OR automation OR robot*) AND (employee* OR worker* OR workforce OR workplace OR occupation* OR professional*) AND (burnout OR "emotional exhaustion" OR depersonalization OR "professional efficacy" OR technostress OR "job stress" OR "work stress" OR wellbeing OR "well-being" OR "work-life balance" OR "job insecurity")) | English; journal article; publication date to 31 March 2026 | Title, abstract, and keyword fields; citation and DOI information retained for deduplication. |
| PubMed/MEDLINE | National Library of Medicine PubMed | (("Artificial Intelligence"[Mesh] OR "artificial intelligence"[Title/Abstract] OR "machine learning"[Title/Abstract] OR "generative AI"[Title/Abstract] OR "large language model"[Title/Abstract] OR "algorithmic management"[Title/Abstract] OR "ambient AI"[Title/Abstract] OR automation[Title/Abstract]) AND (employee*[Title/Abstract] OR worker*[Title/Abstract] OR workplace[Title/Abstract] OR clinician*[Title/Abstract] OR nurse*[Title/Abstract] OR physician*[Title/Abstract]) AND ("Burnout, Professional"[Mesh] OR burnout[Title/Abstract] OR "emotional exhaustion"[Title/Abstract] OR technostress[Title/Abstract] OR "job stress"[Title/Abstract] OR wellbeing[Title/Abstract] OR "well-being"[Title/Abstract] OR "job insecurity"[Title/Abstract])) | English; Humans; database inception to 31 March 2026 | No study-design filter at the search stage; eligibility assessed during screening. |
| APA PsycINFO | APA PsycINFO (EBSCOhost) | TI,AB(("artificial intelligence" OR "machine learning" OR "generative AI" OR GenAI OR "large language model*" OR "algorithmic management" OR "algorithmic control" OR automation OR robot*) AND (employee* OR worker* OR workforce OR workplace OR occupation* OR professional*) AND (burnout OR "emotional exhaustion" OR depersonalization OR technostress OR "job stress" OR "work stress" OR wellbeing OR "well-being" OR "work-life balance" OR "job insecurity")) | Peer-reviewed; English; Human; Journal Article; to 31 March 2026 | Title and abstract fields; peer-reviewed English-language journal articles. |
| Google Scholar | Google Scholar | ("artificial intelligence" OR "generative AI" OR "algorithmic management" OR automation) (employee OR worker OR workplace) (burnout OR "emotional exhaustion" OR technostress OR "job stress" OR wellbeing) | English; searched to 31 March 2026 | Supplementary relevance-ranked searching, followed by backward and forward citation chaining. |
| Backward and forward citation searching | Reference lists and citation indexes | Reference lists of eligible reports and relevant reviews; forward citations of key direct studies | After database screening | Reference lists and cited-by records of eligible studies and relevant reviews. |

**Supplementary Table S2A. Full-text exclusion criteria and coding rules**

One primary exclusion code was assigned to each report. Secondary reasons were retained where relevant but were not double-counted in the study-selection totals.

| **Code** | **Primary reason** | **Operational definition** |
| --- | --- | --- |
| E1 | Non-empirical publication | Review, scoping review, conceptual paper, editorial, commentary or protocol without original participant-level data. |
| E2 | No eligible burnout-related outcome | No burnout, core burnout dimension or prespecified supporting psychological outcome. |
| E3 | No AI-specific workplace exposure | General ICT, digitalization or technology without an identifiable AI, algorithmic, robotic or automation exposure. |
| E4 | Ineligible population or context | Students, patients, consumers or non-working populations; non-workplace setting. |
| E5 | Publication-status ineligible | Grey literature, commercial report, conference proceeding or non-peer-reviewed source where peer review was required. |
| E6 | Duplicate or overlapping report | Duplicate publication or overlapping dataset without additional eligible evidence. |
| E7 | Insufficient empirical reporting | Sample, design, exposure or outcome could not be established sufficiently for extraction. |
| E8 | Full text unavailable | Eligibility could not be assessed after reasonable retrieval attempts. |
| E9 | Language restriction | Full text not available in English. |

**Supplementary Table S2B. Potentially eligible reports excluded after full-text assessment**

Consistent with PRISMA 2020 item 16b, the table identifies reports that might reasonably appear eligible and provides the principal reason for exclusion.

| **No.** | **Report** | **Eligibility-relevant feature** | **Primary exclusion reason** |
| --- | --- | --- | --- |
| 1 | Bankins S, Formosa P, Griep Y, Richards D. Artificial intelligence and the world of work: a review and research agenda. Journal of Organizational Behavior. 2024. | Workplace AI and employee outcomes | E1: Review; no original participant-level data. |
| 2 | Dave B, Martin P, David SS, Kumar S, Chakraborty T. Enhancing healthcare worker mental health via artificial intelligence-driven work process improvements: a scoping review. International Journal of Medical Informatics. 2026;106122. doi:10.1016/j.ijmedinf.2025.106122. | AI and healthcare-worker mental health | E1: Scoping review. |
| 3 | Selenko E, Bankins S, Shoss M, Warburton J, Restubog SLD. Artificial intelligence and the future of work: a functional-identity perspective. Current Directions in Psychological Science. 2022;31(3):272-279. doi:10.1177/09637214221091823. | Identity-threat mechanisms relevant to burnout | E1: Conceptual article. |
| 4 | Zerilli J, Bhatt U, Weller A. How transparency modulates trust in artificial intelligence. Patterns. 2022;3(4):100455. doi:10.1016/j.patter.2022.100455. | Algorithmic transparency | E1/E2: Review and no workplace burnout outcome. |
| 5 | Zirar A, Ali SI, Islam N. Worker and workplace AI coexistence: emerging themes and research agenda. Technovation. 2023;124:102747. doi:10.1016/j.technovation.2023.102747. | Workplace-AI review | E1: Systematic literature review. |
| 6 | Andrei-Barbu I, Pacesila M, Dumitrescu CO. Burnout in the era of artificial intelligence: challenges and benefits. Theoretical and Empirical Researches in Urban Management. 2025;20(4):36-47. | Title appeared directly relevant | E7: Document analysis; no eligible employee exposure-outcome dataset. |
| 7 | Zhao L, Zhao J, Cao E, Li K, Pan L, Zou Y, Sun X. Development and validation of a digital burnout scale in artificial intelligence era. Frontiers in Psychology. 2025;16:1580422. doi:10.3389/fpsyg.2025.1580422. | AI-era burnout measurement | E4: College-student population; not workplace employees. |
| 8 | Gu W, Li M, Zhang S. Algorithmic supervisor and employee performance. Production and Operations Management. 2025;34(10):3101-3118. doi:10.1177/10591478251331095. | Empirical algorithmic supervision | E2: Performance outcomes only; no eligible psychological outcome. |
| 9 | UKG. How AI use can help frontline workers beat burnout. Industry report. 2025. | Workplace AI and self-reported burnout | E5: Commercial, non-peer-reviewed report. |
| 10 | Shchepkina N, Ramnarayan R, Dhaliwal N, Nangia R. Human-centric AI adoption and its influence on worker productivity. BIO Web of Conferences. 2024;86:01060. doi:10.1051/bioconf/20248601060. | Human-centric AI and worker outcomes | E5/E7: Conference proceeding and insufficient reporting for the primary synthesis. |
| 11 | Zhou L, Lv H, Yu Y, et al. An inverted U-shaped relationship between work-family conflict and job burnout among village clinic doctors: the moderating effect of information and communication technology use. BMC Psychiatry. 2024;24:761. doi:10.1186/s12888-024-06221-0. | Burnout and technology-related moderator | E3: ICT use was a moderator; no AI-specific exposure. |
| 12 | Evolahti A, Hultell D, Collins A. Development of burnout in middle-aged working women: a longitudinal study. Journal of Women’s Health. 2013;22(1):94-103. | Longitudinal burnout trajectory | E3: No AI or automation exposure. |
| 13 | Ahola K, Hakanen J, Perhoniemi R, Mutanen P. Relationship between burnout and depressive symptoms: a person-centred study. Burnout Research. 2014;1(1):29-37. | Burnout trajectories | E3: No AI or automation exposure. |
| 14 | Martinent G, Louvet B, Decret JC. Longitudinal trajectories of athlete burnout among young table tennis players: a 3-wave study. Journal of Sport and Health Science. 2020;9(4):367-375. | Dynamic burnout trajectories | E3/E4: No AI exposure and athlete rather than employee population. |
| 15 | Kanayama M, Suzuki M, Yuma Y. Longitudinal burnout-collaboration patterns in Japanese medical care workers. Psychology Research and Behavior Management. 2016:139-146. | Longitudinal occupational burnout | E3: No AI or automation exposure. |
| 16 | Kim B, Lee M, Kim K, Choi H, Lee SM. Longitudinal analysis of academic burnout in Korean middle school students. Stress and Health. 2015;31(4):281-289. | Longitudinal burnout pattern | E3/E4: No AI exposure and student population. |

**Supplementary Table S3A. Direct and core-dimension evidence on AI-related workplace exposure and burnout (n = 20)**

Direct studies measured burnout; core-dimension studies measured an established burnout component, including emotional exhaustion, work exhaustion, or interpersonal disengagement.

| **No.** | **Study / full citation** | **Country, design, and sample** | **AI exposure / role** | **Outcome** | **Reported principal finding** | **Evidence classification** |
| --- | --- | --- | --- | --- | --- | --- |
| 1 | Afshar M, Baumann MR, Resnik F, et al. A pragmatic randomized controlled trial of ambient artificial intelligence to improve health practitioner well-being. NEJM AI. 2025;2(12):AIoa2500945. doi:10.1056/AIoa2500945. | USA; pragmatic stepped-wedge randomized trial; 66 health practitioners | Ambient AI scribe; assistive | Work exhaustion and interpersonal disengagement | Mean change -0.44 (95% CI -0.62 to -0.25; p<.001). | Core burnout dimensions; randomized evidence |
| 2 | Baek G, Cha C. AI-assisted tailored intervention for nurse burnout: a three-group randomized controlled trial. Worldviews on Evidence-Based Nursing. 2025;22(1):e70003. doi:10.1111/wvn.70003. | South Korea; three-group single-blind RCT; 120 nurses | AI-tailored burnout intervention; assistive | Personal, work-related and client-related burnout | Client burnout F=7.725 (p=.001); personal burnout F=10.967 (p<.0001). | Direct burnout evidence; randomized |
| 3 | Chowdhury A, Casey M, Wilson J, et al. Comparing ambient scribes: a randomized crossover clinical trial addressing ambient scribe technologies' impact on physician burnout. Journal of the American Medical Informatics Association. 2026;33(5):990-999. doi:10.1093/jamia/ocag018. | USA; open-label randomized 2 x 2 crossover trial; 160 outpatient clinicians randomized, 136 analyzed | Two ambient AI scribe platforms; assistive | Copenhagen Burnout Inventory, workflow satisfaction, and EHR efficiency | Both tools reduced personal and work-related burnout relative to baseline; between-tool differences in burnout were not meaningful, although product B produced greater workflow satisfaction and a larger reduction in minutes-in-notes. | Direct burnout evidence; randomized crossover trial |
| 4 | Cho A, Cha C, Baek G. Development of an artificial intelligence-based tailored mobile intervention for nurse burnout: single-arm trial. Journal of Medical Internet Research. 2024;26:e54029. doi:10.2196/54029. | South Korea; multiphase single-arm intervention-development study; 300 nurses completed the optimization phase | AI recommender mobile intervention; assistive | Copenhagen Burnout Inventory and job stress | Burnout decreased after the first 2-week program (t=7.012; p<.001) and decreased further after the second program (t=2.811; p=.01); job stress and stress responses also decreased. | Direct burnout evidence; uncontrolled AI-tailored intervention |
| 5 | Olson KD, Meeker D, Troup M, et al. Use of ambient AI scribes to reduce administrative burden and professional burnout. JAMA Network Open. 2025;8(10):e2534976. doi:10.1001/jamanetworkopen.2025.34976. | USA; multicenter pre-post study; 263 clinicians | Ambient AI scribe; assistive | Single-item professional burnout | Burnout decreased from 51.9% to 38.8%; OR=0.26 (95% CI 0.13-0.54). | Direct burnout evidence; pre-post |
| 6 | Misurac J, Knake LA, Blum JM. The effect of ambient artificial intelligence notes on provider burnout. Applied Clinical Informatics. 2025;16(2):252-258. doi:10.1055/a-2461-4576. | USA; pre-post observational study; 38 enrolled, 35 completed | Ambient AI-generated notes; assistive | Stanford Professional Fulfillment Index burnout | Median burnout score decreased from 4.16 to 3.16 (p=.005); prevalence 69% to 43%. | Direct burnout evidence; small pre-post |
| 7 | Liu H, Ding N, Li X, et al. Artificial intelligence and radiologist burnout. JAMA Network Open. 2024;7(11):e2448714. doi:10.1001/jamanetworkopen.2024.48714. | China; nationwide cross-sectional survey; 6,726 radiologists | AI use in radiology; use/perceptual exposure | Maslach Burnout Inventory | AI use was associated with burnout (adjusted OR=1.20, 95% CI 1.10-1.30). | Direct burnout evidence; cross-sectional |
| 8 | Dong J, Zhang G, Wu L. Life against algorithmic management: burnout and its influencing factors among food-delivery riders. Frontiers in Public Health. 2025;13:1531541. doi:10.3389/fpubh.2025.1531541. | China; cross-sectional survey; 953 delivery riders | Algorithmic management; monitoring/control | Maslach Burnout Inventory-General Survey | Multiple algorithmic-management dimensions were positively associated with burnout. | Direct burnout evidence; cross-sectional |
| 9 | Kong H, Yuan Y, Baruch Y, Bu N, Jiang X, Wang K. Influences of artificial intelligence awareness on career competency and job burnout. International Journal of Contemporary Hospitality Management. 2021;33(2):717-734. doi:10.1108/IJCHM-07-2020-0789. | China; cross-sectional SEM; 432 hotel employees | AI awareness; perceptual threat | Job burnout and career competency | AI awareness was positively related to job burnout. | Direct burnout evidence; cross-sectional |
| 10 | Kim BJ, Lee J. The mental health implications of artificial intelligence adoption: the crucial role of self-efficacy. Humanities and Social Sciences Communications. 2024;11:1561. doi:10.1057/s41599-024-04018-w. | South Korea; three-wave survey; 416 professionals | AI adoption; perceptual exposure | Burnout, job stress and AI self-efficacy | No direct AI-burnout effect; significant indirect effect through job stress; self-efficacy buffered the pathway. | Direct burnout evidence; longitudinal mediation |
| 11 | Ali T, Hussain I, Hassan S, Anwer S. Examine how the rise of AI and automation affects job security, stress levels, and mental health in the workplace. Bulletin of Business and Economics. 2024;13(2):1180-1186. doi:10.61506/01.00506. | Pakistan; cross-sectional survey; 300 employees | AI and automation exposure | Job security, stress, anxiety and burnout | AI exposure correlated with burnout (r=.54), stress (r=.72) and anxiety (r=.58); p<.01. | Direct burnout evidence; cross-sectional survey |
| 12 | AlOqaily AN, Qawasmeh EF, Tawalbeh J. The effect of implementing AI on job burnout through the mediating role of work-life balance in HRM. Economics. 2025;13(2):465-484. doi:10.2478/eoik-2025-0049. | Jordan; cross-sectional mediation study; 149 industrial employees | AI implementation in HRM; assistive/organizational | Job burnout and work-life balance | Work-life balance mediated the association between AI implementation and lower burnout. | Direct burnout evidence; cross-sectional |
| 13 | Gao X, Wu W, Guo Y, Lu L. Promotion or suppression? Effect of perceived algorithmic control on service performance of gig workers. Social Behavior and Personality. 2025;53(10):1-13. doi:10.2224/sbp.14812. | China; cross-sectional survey; 475 gig workers | Perceived algorithmic control; monitoring/control | Burnout as mediator; service performance | Burnout mediated the adverse relationship between algorithmic control and performance; transparency weakened the indirect effect. | Direct burnout evidence; cross-sectional |
| 14 | Chuang YT, Chiang HL, Lin AP. Insights from the Job Demands-Resources model: AI’s dual impact on employees’ work and life well-being. International Journal of Information Management. 2025;83:102887. doi:10.1016/j.ijinfomgt.2025.102887. | Three-wave survey; 600 AI-using employees | AI technostress, AI efficacy and generative AI; mixed roles | Exhaustion, engagement, productivity and work-family conflict | AI technostress increased exhaustion; AI efficacy reduced exhaustion; generative AI reduced technostress. | Core burnout-dimension evidence; longitudinal |
| 15 | Thorpe D, Bean C, Krieg J. Associations between generative artificial intelligence usage, job demands, job control and burnout. AI & Society. 2026;41(4):4077-4086. doi:10.1007/s00146-025-02764-2. | Australia; cross-sectional survey; 197 workers | Generative AI use; assistive/perceptual | Burnout, job demands and job control | No statistically significant direct association between generative-AI use and burnout. | Direct burnout evidence; null association |
| 16 | Meduri K, Nadella GS, Gonaygunta H, et al. Human-centered AI for personalized workload management: a multimodal approach to preventing employee burnout. Journal of Infrastructure, Policy and Development. 2024;8(9):6918. doi:10.24294/jipd.v8i9.6918. | Healthcare and IT; cross-sectional survey; 320 workers | Human-centered AI workload management; assistive | Employee burnout/well-being | Training, personalization and feedback were associated with more favorable burnout outcomes. | Direct evidence; quality appraisal required |
| 17 | You JG, Dbouk RH, Landman A, et al. Ambient documentation technology in clinician experience of documentation burden and burnout. JAMA Network Open. 2025;8(8):e2528056. doi:10.1001/jamanetworkopen.2025.28056. | USA; survey studies at two academic health systems; 1,430 clinicians enrolled | Ambient documentation technology; assistive | Professional Fulfillment Index burnout and documentation well-being | Among clinicians completing follow-up assessments, ambient documentation technology was associated with significant reductions in burnout and improved documentation-related well-being; response rates were low. | Direct burnout evidence; survey/pre-post |
| 18 | Stults CD, Deng S, Martinez MC, et al. An ambient artificial intelligence documentation platform for clinicians. JAMA Network Open. 2025;8(5):e258614. doi:10.1001/jamanetworkopen.2025.8614. | USA; pre-post quality-improvement study; 100 clinicians, 57 paired surveys | Ambient AI documentation; assistive | Mini-Z burnout, NASA-TLX and work satisfaction | Burnout decreased from 42.1% to 35.1% but was not statistically significant (p=.12); cognitive load and note time improved. | Direct burnout evidence; non-significant primary change |
| 19 | Zheng J, Zhang T. Association between AI awareness and emotional exhaustion: serial mediation of job insecurity and work interference with family. Behavioral Sciences. 2025;15(4):401. doi:10.3390/bs15040401. | China; cross-sectional survey; 303 private-sector employees | AI awareness; perceptual threat | Emotional exhaustion, job insecurity and work-family interference | AI awareness positively predicted emotional exhaustion; job insecurity and work-family interference serially mediated the association. | Core burnout-dimension evidence |
| 20 | Wang D, Zhou X. The impact of AI awareness on employees’ job burnout: a chain mediation of perceived organizational support and organizational commitment. SAGE Open. 2025;15(4). doi:10.1177/21582440251400532. | China; cross-sectional survey; 434 university teachers | AI awareness; perceptual threat | Job burnout, perceived organizational support and organizational commitment | AI awareness positively predicted burnout; organizational support and commitment formed significant mediating pathways. | Direct burnout evidence; chain-mediation study |

**Supplementary Table S3B. Empirical evidence on mechanisms, proxy outcomes, measurement, and implementation conditions (n = 23)**

Supporting studies inform mechanisms, moderators, implementation conditions, or adjacent occupational psychological outcomes relevant to burnout. Proxy outcomes are not interpreted as equivalent to a validated burnout endpoint.

| **No.** | **Study / full citation** | **Country/design/sample** | **AI exposure / role** | **Outcome or construct** | **Reported principal finding** | **Evidence classification** |
| --- | --- | --- | --- | --- | --- | --- |
| 21 | Albrecht M, Shanks D, Shah T, et al. Enhancing clinical documentation with ambient artificial intelligence: a quality improvement survey assessing clinician perspectives on work burden, burnout, and job satisfaction. JAMIA Open. 2025;8(1):ooaf013. doi:10.1093/jamiaopen/ooaf013. | USA; pre-implementation survey n=93 and post-implementation survey n=99 | Ambient AI documentation; assistive | Documentation burden, perceived burnout risk, and job satisfaction | Post-implementation respondents reported easier documentation, less after-hours work and documentation burden, lower perceived burnout risk, and greater job satisfaction. | Supporting implementation and perceived-risk evidence |
| 22 | Hogemann M, Hein L, Britsche JO, Thomas O. Technostress and generative AI in the workplace: a qualitative analysis of young professionals. Frontiers in Artificial Intelligence. 2025;8:1728881. doi:10.3389/frai.2025.1728881. | Germany; qualitative interviews; 15 young professionals | Generative AI at work; mixed roles | Technostress, dependency, ambiguity and skill degradation | Identified AI-specific stressors and eustress pathways; burnout was not directly measured. | Supporting mechanism evidence |
| 23 | Issa H, Jaber J, Lakkis H. Navigating AI unpredictability: exploring technostress in AI-powered healthcare systems. Technological Forecasting and Social Change. 2024;202:123311. doi:10.1016/j.techfore.2024.123311. | Healthcare AI users; cross-sectional survey; 224 | AI unpredictability; demand | Technostress/eustress-distress | Techno-unpredictability generated both distress and eustress depending on appraisal and coping. | Supporting mechanism evidence |
| 24 | Koch M, Lodefalk M. Artificial intelligence and worker stress: evidence from Germany. Digital Society. 2025;4:5. doi:10.1007/s44206-025-00160-3. | Germany; repeated cross-sectional worker surveys; approximately 20,000 per wave | AI/robot exposure; automation/assistive | Worker stress | AI/robot exposure was not associated with higher stress and was negatively associated with increased stress in several models. | Supporting proxy evidence |
| 25 | Braganza A, Chen W, Canhoto A, Sap S. Productive employment and decent work: the impact of AI adoption on psychological contracts, job engagement and employee trust. Journal of Business Research. 2021;131:485-494. doi:10.1016/j.jbusres.2020.08.018. | UK/Turkey; employee survey; 232 | Organizational AI adoption | Psychological contract, engagement and trust | AI adoption altered psychological-contract relationships with engagement and trust. | Supporting contextual evidence |
| 26 | Gull A, Ashfaq J, Aslam M. AI in the workplace: impact on employee well-being and cognitive job insecurity. International Journal of Business and Economic Affairs. 2023;8(4):79-91. doi:10.24088/IJBEA-2023-84007. | Pakistan; time-lagged employee survey; 253 | AI identity threat/awareness | Cognitive job insecurity and employee well-being | AI-related identity threat increased cognitive job insecurity, which was associated with poorer well-being. | Supporting mechanism evidence |
| 27 | Nazareno L, Schiff DS. The impact of automation and artificial intelligence on worker well-being. Technology in Society. 2021;67:101679. doi:10.1016/j.techsoc.2021.101679. | USA; General Social Survey 2002-2018 linked to 402 occupations | Automation/AI occupational risk | Stress, health and job satisfaction | Higher automation risk was associated with lower immediate stress but poorer health and less favorable job satisfaction. | Supporting proxy evidence |
| 28 | Stamate AN, Sauve G, Denis PL. The rise of the machines and how they impact workers’ psychological health: an empirical study. Human Behavior and Emerging Technologies. 2021;3(5):942-955. doi:10.1002/hbe2.315. | Canada; employee survey/path analysis; 546 | Perceived usefulness of AI/automation | Basic-needs satisfaction/thwarting and psychological health | AI perceptions related to psychological health through basic-needs satisfaction and thwarting. | Supporting well-being evidence |
| 29 | Paya Castiblanque R, Pizzi A. Relationship between certain uses of artificial intelligence and psychosocial risk factors in European work environments. Archivos de Prevencion de Riesgos Laborales. 2024;27(3):233-249. doi:10.12961/aprl.2024.27.03.02. | European Union; cross-sectional survey; 27,252 workers | AI monitoring/task allocation; monitoring/control | Time pressure, autonomy and stress-related outcomes | AI monitoring was associated with time pressure/overload, reduced autonomy and stress-related outcomes. | Supporting monitoring evidence |
| 30 | Mendez-Suarez M, Cukusic M, Nincevic-Pasalic I. AI FoMO (fear of missing out) in the workplace. Technology in Society. 2026;84:103052. doi:10.1016/j.techsoc.2025.103052. | OECD workplace-AI data; configurational/fsQCA analysis | AI-related fear of missing out | Skill devaluation, autonomy loss and mental-health risk | Configurations involving skill devaluation, autonomy loss and AI supervision were linked to AI FoMO. | Supporting mechanism evidence |
| 31 | Zheng J, Zhang JZ, Kamal MM, Liang X, Alzeiby EA. Unpacking human-AI interaction: unintended consequences on employee well-being in entrepreneurial firms. Journal of Business Research. 2025;196:115406. doi:10.1016/j.jbusres.2025.115406. | Comparative longitudinal case study; six high-tech ventures | AI-enabled HRM and human-AI interaction | Employee well-being and unintended consequences | Identified autonomy erosion, surveillance precarity, bias and personalized discontentment as AI-HRM risks. | Supporting longitudinal/contextual evidence |
| 32 | Kumar A, Krishnamoorthy B, Bhattacharyya SS. Machine learning and artificial intelligence-induced technostress in organizations: automation-augmentation paradox and socio-technical coping. International Journal of Organizational Analysis. 2024;32(4):681-701. doi:10.1108/IJOA-01-2023-3581. | Qualitative interviews with managers; sample NR | AI/ML automation-augmentation paradox | Technostress and socio-technical coping | Explained how automation and augmentation can jointly generate technostress and how socio-technical design may buffer it. | Supporting qualitative evidence |
| 33 | Zhang R, Liu Y, Qasim M, Wang X. More than a tool: how generative AI shapes employees’ work-goal progress. Personnel Review. 2026;55(1):418-454. doi:10.1108/PR-05-2025-0478. | Three-phase field study plus experiment | Generative AI use; assistive/relational | Work-goal progress, resource gains/losses and emotional labor | Generative AI produced both resource gains and relational/emotional costs affecting goal progress. | Supporting mechanism evidence |
| 34 | Kong H, Yin Z, Baruch Y, Yuan Y. The impact of trust in AI on career sustainability: the role of employee-AI collaboration and protean career orientation. Journal of Vocational Behavior. 2023;146:103928. doi:10.1016/j.jvb.2023.103928. | Two studies; Study 2 included 447 employee-supervisor dyads | Trust in AI and employee-AI collaboration; resource | Career sustainability, well-being and productivity | Trust in AI predicted career sustainability and well-being through employee-AI collaboration. | Supporting resource evidence |
| 35 | Duong NH, Hung HN, An NH, et al. Psychological drivers of employee-AI collaboration and career sustainability: the role of trust, threat and protean orientation. Acta Psychologica. 2026;262:106074. doi:10.1016/j.actpsy.2025.106074. | Online employee survey; 532 | AI trust/threat and employee-AI collaboration | Career sustainability | AI trust positively predicted collaboration; motivational pathways linked threat appraisal to collaboration and career sustainability. | Supporting psychological-resource evidence |
| 36 | Wu W, Yang Y, Yang L, Su L, Chen Z. The inverted U-shaped relationship between AI and job satisfaction: evidence from China’s labor-force data. Emerging Markets Finance and Trade. 2025. doi:10.1080/1540496X.2025.2548427. | China Labor-force Dynamic Survey secondary analysis; sample NR | AI exposure/intensity | Job satisfaction | AI showed an inverted U-shaped association with job satisfaction; the turning point varied by skill level. | Supporting nonlinear proxy evidence |
| 37 | Arboh F, Zhu X, Atingabili S, Yeboah E, Drokow EK. From fear to empowerment: the impact of employees’ AI awareness on workplace well-being. Journal of Health Organization and Management. 2025. doi:10.1108/JHOM-06-2024-0229. | Health workers; empirical survey; sample NR | Employee AI awareness | Workplace well-being and informal learning | AI awareness showed demand and resource pathways to well-being through informal learning and appraisal. | Supporting well-being evidence |
| 38 | Jin G, Jiang J, Liao H. The work affective well-being under the impact of AI. Scientific Reports. 2024;14:25483. doi:10.1038/s41598-024-75113-w. | China; employee questionnaire study; 349 | STARA/AI awareness; perceptual threat | Work affective well-being, job stress and resilience | STARA awareness negatively predicted well-being; job stress mediated and resilience buffered the pathway. | Supporting well-being evidence |
| 39 | Chung YW, Im S, Kim JE, Yun JK. Artificial intelligence awareness, career resilience, job insecurity and behavioural outcomes. Australian Journal of Psychology. 2025;77(1):2559910. doi:10.1080/00049530.2025.2559910. | South Korea; three-wave survey of full-time office employees; sample NR | AI awareness; perceptual threat | Job insecurity, task performance and deviant behavior | Job insecurity mediated AI-awareness effects; career resilience buffered the AI-awareness-to-insecurity association. | Supporting mechanism evidence |
| 40 | Zhou Y, Lyu B. How does leadership AI awareness shape employee voice behavior? Based on the framework of hindrance and challenge stressors. Work. 2025;82(1):289-305. doi:10.1177/10519815251341816. | China; survey of 487 employees in knowledge-intensive industries | Leadership AI awareness | Job insecurity and employee voice | Job insecurity mediated a negative pathway from leadership AI awareness to voice behavior. | Supporting mechanism evidence |
| 41 | Cheng WJ, Pien LC, Cheng Y. Occupation-level automation probability is associated with psychosocial work conditions and workers’ health: a multilevel study. American Journal of Industrial Medicine. 2021;64(2):108-117. doi:10.1002/ajim.23210. | Taiwan; national survey; 14,948 workers | Occupation-level automation probability | Burnout, job insecurity, work demands/control and worker health | Automation probability was associated with psychosocial conditions and health; burnout patterns differed across automation-risk groups. | Supporting automation evidence; indirect AI exposure |
| 42 | Brougham D, Haar J. Smart Technology, Artificial Intelligence, Robotics, and Algorithms (STARA): employees’ perceptions of our future workplace. Journal of Management & Organization. 2018;24(2):239-257. doi:10.1017/jmo.2016.55. | Mixed-methods study; 120 employees | STARA awareness; perceptual threat | Commitment, career satisfaction, turnover intentions, cynicism and depression | Greater STARA awareness was associated with poorer career/well-being outcomes and greater cynicism/depression. | Supporting foundational evidence |
| 43 | Lingmont DNJ, Alexiou A. The contingent effect of job automating technology awareness on perceived job insecurity: the moderating role of organizational culture. Technological Forecasting and Social Change. 2020;161:120302. doi:10.1016/j.techfore.2020.120302. | Cross-sectional employee survey; 291 participants | STARA/job-automation awareness | Perceived job insecurity; organizational culture | STARA awareness positively predicted job insecurity; authoritarian culture strengthened the relationship. | Supporting mechanism evidence |

**Supplementary Table S4A. MMAT 2018 design-specific appraisal criteria**

| **Code** | **Design category** | **C1** | **C2** | **C3** | **C4** | **C5** |
| --- | --- | --- | --- | --- | --- | --- |
| QUAL | Qualitative | Approach appropriate | Data collection adequate | Findings derived from data | Interpretation substantiated | Coherence across data, analysis, and interpretation |
| Q-RCT | Randomized controlled | Randomization appropriate | Groups comparable at baseline | Outcome data complete | Outcome assessors blinded | Adherence to intervention |
| Q-NR | Quantitative non-randomized | Participants representative | Measurements appropriate | Outcome data complete | Confounders accounted for | Exposure/intervention as intended |
| Q-D | Quantitative descriptive | Sampling strategy relevant | Sample representative | Measurements appropriate | Low nonresponse bias | Statistical analysis appropriate |
| MM | Mixed methods | Rationale appropriate | Components effectively integrated | Integrated outputs interpreted | Divergences addressed | Components meet quality criteria |

**Supplementary Table S4B. Study-level MMAT 2018 appraisal of the 43 included primary studies**

S1: clear research question or objective. S2: collected data address the research question or objective. Design-specific judgments are reported at criterion level; no aggregate numerical quality score was calculated. CT indicates insufficient reporting for a definitive judgment.

| **No.** | **Study** | **Design** | **S1** | **S2** | **C1** | **C2** | **C3** | **C4** | **C5** | **Key appraisal note** |
| --- | --- | --- | --- | --- | --- | --- | --- | --- | --- | --- |
| 1 | Afshar et al. (2025) | Q-RCT | Y | Y | Y | Y | Y | CT | Y | Stepped-wedge randomized design; assessor blinding not clearly reported. |
| 2 | Baek & Cha (2025) | Q-RCT | Y | Y | Y | Y | Y | CT | Y | Random allocation and baseline comparisons reported; blinded outcome assessment unclear. |
| 3 | Chowdhury et al. (2026) | Q-RCT | Y | Y | Y | Y | CT | N | Y | Randomized crossover allocation and baseline assessment were reported; 136 of 160 randomized clinicians were analyzed, and the open-label self-reported outcomes precluded assessor blinding. |
| 4 | Cho et al. (2024) | Q-NR | Y | Y | CT | Y | Y | N | Y | Multiphase single-arm intervention used validated burnout measures; the absence of a control group limits confounding control. |
| 5 | Olson et al. (2025) | Q-NR | Y | Y | CT | Y | Y | N | Y | Multicenter pre-post evaluation; no concurrent control group. |
| 6 | Misurac et al. (2025) | Q-NR | Y | Y | CT | Y | Y | N | Y | Validated outcome measure; small uncontrolled pre-post design without confounder adjustment. |
| 7 | Liu et al. (2024) | Q-D | Y | Y | Y | Y | Y | CT | Y | Large nationwide sample and validated burnout measure; nonresponse bias not fully resolved. |
| 8 | Dong et al. (2025) | Q-D | Y | Y | CT | CT | Y | CT | Y | Relevant rider sample and validated burnout measure; representativeness not fully established. |
| 9 | Kong et al. (2021) | Q-D | Y | Y | CT | CT | Y | CT | Y | Cross-sectional SEM with established constructs; convenience-sampling risk. |
| 10 | Kim & Lee (2024) | Q-NR | Y | Y | CT | Y | Y | Y | Y | Three-wave design and indirect-effect testing; causal inference remains limited. |
| 11 | Ali et al. (2024) | Q-D | Y | Y | CT | CT | Y | CT | Y | Cross-sectional survey; sampling and nonresponse information limited. |
| 12 | AlOqaily et al. (2025) | Q-D | Y | Y | CT | CT | Y | CT | Y | Online survey and PLS mediation; representativeness and nonresponse require caution. |
| 13 | Gao et al. (2025) | Q-D | Y | Y | CT | CT | CT | Y | Y | Cross-sectional moderated mediation; measurement details require full-report interpretation. |
| 14 | Chuang et al. (2025) | Q-NR | Y | Y | CT | Y | Y | Y | Y | Three-wave design with temporally separated measures; residual confounding remains possible. |
| 15 | Thorpe et al. (2026) | Q-D | Y | Y | CT | CT | Y | CT | Y | Cross-sectional survey; null association interpreted without causal claims. |
| 16 | Meduri et al. (2024) | Q-D | Y | Y | CT | CT | CT | CT | Y | Quantitative survey; detailed sampling and measurement reporting should be interpreted cautiously. |
| 17 | You et al. (2025) | Q-NR | Y | Y | CT | Y | CT | N | Y | Two-system implementation study used established burnout measures; low follow-up response and lack of a concurrent control limit inference. |
| 18 | Stults et al. (2025) | Q-NR | Y | Y | Y | Y | CT | N | Y | Purposive specialty sampling and paired outcomes were reported; attrition and the uncontrolled design remain limitations. |
| 19 | Zheng & Zhang (2025) | Q-NR | Y | Y | CT | Y | Y | Y | Y | Employee survey used validated measures and serial mediation with covariate-adjusted regression; the design was cross-sectional. |
| 20 | Wang & Zhou (2025) | Q-NR | Y | Y | CT | Y | Y | Y | Y | Validated measures and chain mediation were reported for 434 full-time university teachers; causal direction cannot be established. |
| 21 | Albrecht et al. (2025) | Q-NR | Y | Y | CT | CT | CT | N | Y | Pre- and post-surveys were not paired and burnout risk was assessed by perception rather than a validated burnout instrument. |
| 22 | Hogemann et al. (2025) | QUAL | Y | Y | Y | Y | Y | Y | Y | Coherent qualitative design and analysis; small purposive sample. |
| 23 | Issa et al. (2024) | Q-D | Y | Y | CT | CT | Y | CT | Y | Cross-sectional technostress study; self-report and sampling limitations. |
| 24 | Koch & Lodefalk (2025) | Q-D | Y | Y | Y | Y | Y | CT | Y | Large repeated cross-sectional datasets; exposure measurement is occupation/technology based. |
| 25 | Braganza et al. (2021) | Q-D | Y | Y | CT | CT | Y | CT | Y | Employee survey; cross-sectional self-report design. |
| 26 | Gull et al. (2023) | Q-NR | Y | Y | CT | Y | Y | Y | Y | Time-lagged design; sampling representativeness remains uncertain. |
| 27 | Nazareno & Schiff (2021) | Q-D | Y | Y | Y | Y | Y | CT | Y | National data linked to occupational automation risk; exposure is indirect. |
| 28 | Stamate et al. (2021) | Q-D | Y | Y | CT | CT | Y | CT | Y | Cross-sectional path analysis; self-report and sampling limitations. |
| 29 | Paya Castiblanque & Pizzi (2024) | Q-D | Y | Y | Y | Y | Y | CT | Y | Large EU dataset; cross-sectional associations and proxy outcomes. |
| 30 | Mendez-Suarez et al. (2026) | Q-D | Y | Y | Y | CT | Y | CT | Y | OECD data and fsQCA appropriate to configurational question; sample details should be read with source documentation. |
| 31 | Zheng et al. (2025) | QUAL | Y | Y | Y | Y | Y | Y | Y | Longitudinal comparative case design with coherent qualitative interpretation. |
| 32 | Kumar et al. (2024) | QUAL | Y | Y | Y | CT | Y | Y | Y | Qualitative design appropriate; sample details limited in accessible reporting. |
| 33 | Zhang et al. (2026) | MM | Y | Y | Y | Y | Y | CT | Y | Multiphase field and experimental evidence; integration reporting partly unclear. |
| 34 | Kong et al. (2023) | Q-NR | Y | Y | CT | Y | Y | Y | Y | Two-study program included multisource two-wave data from 447 employee-supervisor dyads and appropriate moderated mediation tests. |
| 35 | Duong et al. (2026) | Q-NR | Y | Y | CT | Y | CT | Y | Y | Online employee survey and PLS-SEM addressed the stated model; representativeness and nonresponse were not fully established. |
| 36 | Wu et al. (2025) | Q-D | Y | Y | Y | Y | Y | CT | Y | Large secondary dataset; job satisfaction is a proxy rather than burnout. |
| 37 | Arboh et al. (2025) | Q-NR | Y | Y | CT | Y | CT | Y | Y | Empirical health-worker survey addressed AI awareness and workplace well-being; sampling and response information were incompletely reported. |
| 38 | Jin et al. (2024) | Q-NR | Y | Y | CT | Y | Y | Y | Y | Employee questionnaire study reported screening, validated scales, controls, and mediation/moderation analyses; causal inference remains limited. |
| 39 | Chung et al. (2025) | Q-NR | Y | Y | CT | Y | Y | Y | Y | Three-wave employee survey reduced same-time measurement bias; attrition from 300 invitations to 209 analyzed cases should be considered. |
| 40 | Zhou & Lyu (2025) | Q-NR | Y | Y | CT | Y | CT | Y | Y | Survey of 487 employees tested mediation and stressor pathways; sampling and nonresponse information were not fully reported. |
| 41 | Cheng et al. (2021) | Q-NR | Y | Y | Y | Y | Y | Y | Y | National random worker sample, multilevel exposure assignment, established outcomes, and adjusted analyses supported the study question. |
| 42 | Brougham & Haar (2018) | MM | Y | Y | Y | Y | Y | CT | Y | Mixed-methods design developed STARA awareness and linked it to multiple well-being outcomes; handling of divergent findings was not fully clear. |
| 43 | Lingmont & Alexiou (2020) | Q-NR | Y | Y | CT | Y | Y | Y | Y | Employee survey used established constructs and moderation tests; representativeness of high-automation industries remains uncertain. |
